# Recovery from a Poisonous Dose of Strychnia

**Published:** 1848-07

**Authors:** 


*Recovery from a poisonous dose of Strychnia.*—Dr. Thomas Anderson records, in the *Monthly Journal and Retrospect of the Medical Sciences*, a case in which seven grains of strychnia were taken without producing fatal consequences. The subject of the case was a gentleman who had long suffered from tic douloureux, for the relief of which he was in the habit of taking muriate of morphia, in increased doses, until three and a half grains were necessary to produce its effect. Having occasion to go a short distance in the country on business, he took, previous to setting out, his usual dose, three and a half grains of strychnine, given him by an apothecary by mistake for muriate of morphia, which he placed on his tongue and swallowed;—he remarked at the time that it was extremely bitter, and that the taste was more than usually persistent, but it did not occur to him that anything was wrong. Shortly after, however, while walking along the street, he felt slightly indisposed, the most prominent symptom being a sense of numbness in the back of the legs, which he attributed to the effects of cold, to which he had been exposed in the early part of the morning. As these symptoms did not appear of any importance, he proceeded by a public conveyance to the village where his business lay, and returned by the next opportunity. During the whole of this time the symptoms remained precisely as they were the moment he first observed them; but as he was walking along, on his return, they suddenly increased, the numbness being accompanied by a sense of want of power, and a sort of dragging of the muscles of the legs, which soon became so great, that, as he describes it, he had to put his hands at the back of his thighs in order to push his legs along. This occurred at nearly two and a half hours after he had taken the dose of the supposed muriate of morphia, and, at this time, there could have been nothing remarkable or unusual in his appearance; for, on his way home, he met a friend to whom he communicated his sensations, but who laughed at his evident apprehension, and assured him that it was all imagination. As he was in the midst of describing the effect upon his muscles, and bending himself so as to show how it occurred, he suddenly overbalanced and fell heavily backwards. He was immediately raised, and, on attaining the upright position, he felt himself much in the same state in which he was before, excepting that he was excessively nervous and alarmed. The want of power in the legs did not at all increase in intensity, and no spasmodic affection was observed, although the patient was himself under the impression, that his fall was somehow connected with the previous symptoms.

The patient's fall and the nervous state into which he had got, now fairly alarmed his friend, who begged him to get home as fast as possible, and accompanied him on his way, as he experienced considerable difficulty in walking, and could not get on without support. On reaching home he felt somewhat better, and remained sitting for some time, and at length retired to bed, about five hours after the first appearance of the symptoms. Just previous to stepping into bed, in order to insure a good night's rest, of which the recent symptoms rendered him somewhat doubtful, he took a second dose of the powder, equal in amount to the first. In less than ten minutes after, he was seized with a violent tetanic spasm, affecting the legs and muscles of respiration, and had only time to call out for assistance before the sensation amounted to that of absolute suffocation. Fortunately, assistance was close at hand, and he was immediately raised up in bed, with the effect of entirely relieving the sense of suffocation, and a medical man sent for. Spasms now followed each other in rapid succession, the intervals being about a quarter of an hour or twenty minutes, and the affection was confined principally to the legs, back, and respiratory muscles, the arms being comparatively unaffected. The numbness and dragging of the muscles, which had been continuous during the first five hours, disappeared entirely during the intervals of the spasms, and the patient was left without any uneasy sensations, excepting the exhaustion of the previous fits and the apprehension of its successor. During the whole of this time he was not only perfectly conscious, but his senses were preternaturally exalted, and he distinctly heard a variety of whispered observations of the physicians and his friends, which, from their tenor, were obviously not intended to reach the ears of the patient.

The paroxysms, after continuing for some time, began gradually to diminish in violence, the intervals becoming longer, and the duration of each spasm shorter, and it was hoped that they were about to pass off, when all at once they returned in their original violence. This proved, however, to be the last expiring effort of the poison; for the symptoms now entirely ceased, about thirteen hours after the first dose was taken. At the conclusion of the spasms, the patient was left in an excessively exhausted state, and was unable to turn himself in bed; from this, however, he recruited with great rapidity, as he was able to get up on the evening of the next day, and on the second he walked out and went about his usual business. The most remarkable fact connected with the case is, that, from that time the attacks of tic douloureux entirely ceased, and he has not since had any return of it. The medical treatment employed in the case was unimportant, and had not any effect on the progress of the symptoms.

